# Prediction of Mortality and Postoperative Complications using the Hip-Multidimensional Frailty Score in Elderly Patients with Hip Fracture

**DOI:** 10.1038/srep42966

**Published:** 2017-02-24

**Authors:** Jung-Yeon Choi, Kwan-Jae Cho, Sun-wook Kim, Sol-Ji Yoon, Min-gu Kang, Kwang-il Kim, Young-Kyun Lee, Kyung-Hoi Koo, Cheol-Ho Kim

**Affiliations:** 1 Department of Internal Medicine, Seoul National University College of Medicine, Seoul, Republic of Korea; 2 Department of Orthopedic Surgery, Seoul National University College of Medicine, Seoul, Republic of Korea; 3 Department of Internal Medicine, Seoul National University Bundang Hospital, Seongnam, Republic of Korea; 4 Department of Orthopedic surgery, Seoul National University Bundang Hospital, Seongnam, Republic of Korea

## Abstract

High mortality and dependent living after hip fracture pose a significant public health concern. Retrospective study was conducted with 481 hip fracture patients (≥65 years of age) undergoing surgery from March 2009 to May 2014. The Hip-MFS was calculated by Comprehensive Geriatric Assessment (CGA). The primary outcome was the 6-month all-cause mortality rate. The secondary outcomes were 1-year all-cause mortality, postoperative complications and prolonged hospital stay, and institutionalization. Thirty-five patients (7.3%) died within 6 months after surgery (median [interquartile range], 2.9 [1.4–3.9] months). The fully adjusted hazard ratio per 1 point increase in Hip-MFS was 1.458 (95% confidence interval [CI]: 1.210–1.758) for 6-months mortality and odds ratio were 1.239 (95% CI: 1.115–1.377), 1.156 (95% CI: 1.031–1.296) for postoperative complications and prolonged total hospital stay, respectively. High-risk patients (Hip-MFS > 8) showed higher risk of 6-month mortality (hazard ratio: 3.545, 95% CI: 1.466–8.572) than low-risk patients after adjustment. Hip-MFS successfully predict 6-month mortality, postoperative complications and prolonged hospital stay in elderly hip fracture patients after surgery. Hip-MFS more precisely predict 6-month mortality than age or existing tools (P values of comparison of ROC curve: 0.002, 0.004, and 0.044 for the ASA classification, age and NHFS, respectively).

Approximately 12.1% of women and 4.6% of men at age 50 years are predicted to experience hip fracture in their lifetime in Western countries^1^. Furthermore, 87–96% of hip fracture patients are 65 years of age or older^2^,^3^.

Hip fractures pose a significant public health concern because of reduced life expectancy by 1.8 years and substantial socioeconomic effects with high institutionalization rate of approximately 30% at 6 months after surgery^4^,^5^,^6^. If a prognosis could be assessed at the time of admission, a better allocation of limited resources could lead to improved outcomes and efficient postoperative care.

Frailty, defined as a consequence of a reduction in physiologic reserves, is a major contributor to poor surgical outcomes in geriatric patients^7^. Previous studies demonstrated that patients with decreased cognitive function, higher comorbidity, intra-capsular fracture, and prolonged postoperative delirium were susceptible to mortality after hip fracture surgery^8^,^9^. However, there is a scarcity of grading systems including the concept of frailty to predict postoperative outcomes.

In a recent study, the risk of in-hospital mortality and major postoperative complications was higher after hip fracture surgery than after elective total hip replacement after matching age, sex, and preoperative medical conditions^10^. Although fracture-related acute physiologic processes could influence the additional risk, hip fracture patients tend to be much frailer than total hip replacement patients. Frailty could be insufficiently adjusted by matching only for age, sex, and preoperative medical conditions.

In our previous studies, the Multidimensional Frailty Score (MFS), derived from the comprehensive geriatric assessment (CGA) with the concept of cumulative deficit and frailty, predicted adverse outcomes in patients undergoing intermediate to high-risk elective operations and in female patients who were designated American Society of Anesthesiologists (ASA) physical status class 1 or 2 and underwent cancer surgery^11^,^12^.

Thus, we aimed to identify the predictive value of a modified model of the MFS (Hip-MFS) to predict mortality, postoperative complications, institutionalization and prolonged hospital stay in elderly hip fracture patients.

## Methods

### Study Population

This retrospective cohort study was conducted at Seoul National University Bundang Hospital, a 1300-bed teaching hospital. Patients aged 65 years or older who planned to undergo hip fracture surgery were referred to multidisciplinary geriatric teams for CGA. The CGA were conducted before discharge and within a month before or after the surgery, with the exception of 10 patients evaluated 1 to 3 months before surgery. From March 2009 to May 2014, 1075 elderly hip fracture patients underwent surgery and 538 patients received CGA before discharge. 57 patients were excluded: 5 who were treated conservatively, 5 who refused surgery, 2 who died before surgery, and 45 patients with missing data to calculate the Hip-MFS. As a result, 481 patients were included in our final analysis. Baseline characteristics of the patients, including age, anthropometric data and ASA classification were retrieved from electronic medical records.

The study protocol was reviewed and approved by the Seoul National University Bundang Hospital institutional review board, which waived the requirement for informed consent. In addition to this, all methods were performed in accordance with the Strengthening the reporting of observational studies in epidemiology Statement and regulation of the institutional review board.

### Hip-Multidimensional Frailty Score

The MFS was amended for hip fracture patients by including sex, risk of falling, and walking dependency. Comorbidity was evaluated by the Charlson comorbidity index, a weighted index that considers the number and seriousness of comorbid diseases based on the risk of 1-year mortality^13^. Physical function was estimated according to walking dependency by the Koval grade^14^. Cognitive function was assessed by the Korean version of the Mini-Mental State Examination (MMSE-KC), and depression was evaluated with the Korea Geriatric Depression Scale^15^,^16^. Risk of malnutrition was analysed using the Mini Nutritional Assessment, which was designed and validated to provide a rapid assessment of nutrition status in elderly patients^17^. The risk of falling was assessed by the Predisposition for Falling assessment guide, which measures 10 factors (age, mental status, number of days in hospital, bowel and bladder incontinence, history of falling, uncompensated visual impairment, ambulation status, drop in systolic blood pressure, gait and balance disturbance, and classifications of medication), with a score of 10 or above indicating a risk of falling^18^.

Hip-MFS is composed of 8 domains (serum albumin level, mid-arm circumference, Charlson comorbidity index, walking dependency, cognitive function, risks of falling, nutritional status, and sex). We used reference cut-off values from previous studies for 5 of the 8 domains (serum albumin level, mid-arm circumference, Charlson comorbidity index, cognitive function, and nutritional status)^11^,^12^. The patients who were diagnosed with dementia or could not complete the MMSE-KC test because of decreased cognitive function were categorized as score 2 for the cognitive function domain of Hip-MFS. The risks of falling were scored (1 or 0) according to the well-established reference value (score of 10 or above). Male gender was converted to score 1 and female gender was converted to score 0 according to the preceding meta-analysis of preoperative indicators for mortality following hip fracture surgery^3^,^5^,^8^. The patient’s walking dependency was categorized as independence (Koval grade 1), limited independence (Koval grade 2 to 6), and unable to walk (Koval grade7) and converted to score 0, 1, and 2, respectively^19^. The Hip-MFS ranged from 0–14, with higher scores corresponding to greater frailty (Table 1).

### Outcomes

The primary endpoint was 6-month all-cause mortality after surgery. The data of all deaths obtained by the Ministry of Security and Public Administration occurred through July 15, 2015. The secondary outcomes were 1-year all-cause mortality, postoperative complications, institutionalization, and prolonged total or postoperative hospital stay. We defined 6-month all-cause mortality instead of 1-year all-cause mortality to identify high-risk patients expected to have short-term adverse outcomes and to emphasize more intensive perioperative care for those patients.

Postoperative complications included pneumonia, urinary tract infection, delirium, pulmonary thromboembolism, deep vein thrombosis, stroke, and unplanned intensive care unit (ICU) admission. Delirium was diagnosed by the DSM-IV criteria. The standard National Surgical Quality Improvement Program definitions were used to diagnose pneumonia, urinary tract infection, pulmonary thromboembolism, deep vein thrombosis, and stroke^20^. Unplanned ICU admission was specified as entering a medical or surgical ICU at least 72 hours after surgery for close monitoring of cardiopulmonary instability, renal failure, infection, or bleeding. A discharge to a nursing home, transitional care facility, or any long-term care centres was defined as institutionalization. Delayed surgery was defined surgery performed as more than 120 hours after admission according to the previous cut-off value influencing mortality^21^. Predictive value of Hip-MFS was also analysed both early and delay surgery subgroup.

### Statistical Analysis

Statistical analysis was performed using SPSS version 21.0 (SPSS Inc., Chicago, IL, USA) and StataSE version13 (Stata Corporation, College Station, TX, USA). Continuous variables are expressed as mean (standard deviation) or median (interquartile range [IQR]) for variables not normally distributed. Continuous variables were compared using unpaired *t*-tests. Categorical variables are represented as number or proportion, and the χ^2^ or Fisher’s exact test was used to compare proportions.

The effects of Hip-MFS on postoperative complication and prolonged hospital stay were evaluated by logistic regression analysis. Full adjustment was made with variables such as age, body mass index, white blood cell count, haemoglobin, total cholesterol, total protein, serum creatinine level, blood urea nitrogen, and ASA classification. Prolonged total or postoperative hospital stay was defined as the 75^th^ percentile cut-off for total (>28 days) or postoperative (>23 days) hospital stay. Kaplan-Meier analysis was used for survival curves, and log-rank tests were used to assess significance. Cox’s proportional hazard analysis was used to estimate the hazard ratios (HRs) for mortality. We compared the model’s predictive value for the primary outcome with chronological age, ASA classification, and Nottingham Hip Fracture Score (NHFS) using comparison of ROC curve^22^. The ASA class indicates an individual’s physical health and predicts post-operative morbidity ranging from 0 (lowest risk) to 6 (highest risk)^23^. NHFS is a weighted score of seven independent admission variables which reliably predicts 30 day mortality for patients after hip fracture^22^.

Repeated measures ANOVA was used for comparing the preoperative and postoperative Koval grade between Hip-MFS groups. All calculated *P*-values were 2-tailed, with values <0.05 considered statistically significant.

## Results

### Study Population

Of 481 patients, the median (IQR) age of patients was 80.4 (75.3–85.3) years. The median length of total hospital stay and postoperative hospital stay were 18 (11–28) days, and 14 (8–23) days, respectively. A total of 183 patients (38.0%) experienced at least 1 postoperative complication including pneumonia (21 patients), urinary tract infection (20 patients), pulmonary thromboembolism (4 patients), deep vein thrombosis (5 patients), delirium (162 patients), stroke (3 patients), and unplanned ICU admission (18 patients), and 282 patients (58.6%) were discharged to transitional care facilities or nursing homes. Nineteen patients experienced 2 complications, 12 patients had 3 complications, and 1 patient each had 4 and 5 complications, respectively. The median follow-up period was 34.6 (19.7–52.4) months. Overall mortality was 1.2% at 1 month, 7.3% at 6 months, 13.9% at 1 year and 34.9% at the end of follow-up.

Low haemoglobin, low serum protein levels, low serum albumin levels, high serum creatinine levels, and ASA classification were associated with increased 6–month all-cause mortality. Among the geriatric assessment domains, a low MMSE-KC score, dependency in IADL, a higher Charlson comorbidity index, risk of falling, malnutrition, and thinner mid-arm circumference were associated with increased 6-month all-cause mortality (Table 2).

### Hip-Multidimensional Frailty Score and Outcomes

The patients were categorized into high-risk (Hip-MFS > 8) and low-risk (Hip-MFS ≤ 8) groups with respect to the receiver operating characteristic curve of the primary outcome and the Youden index^24^. The sensitivity and specificity for predicting 6-month all-cause mortality rates were 62.9% and 78.7%, respectively, according to the model’s cut-off point (>8 vs. ≤8).

The hazard ratios per 1 point in the model were 1.458 (95% CI: 1.210–1.758, *P* < 0.001) for 6-month all-cause mortality, 1.419 (95% CI: 1.239–1.626, *P* < 0.001) for 1-year all-cause mortality after full adjustment. The odds ratios per 1 point in the model were 1.239 (95% CI: 1.115–1.377, *P* < 0.001) for postoperative complications, 1.078 (95% CI: 0.978–1.191, *P* = 0.131) for institutionalization, 1.187 (95% CI: 1.053–1.339, *P* = 0.028) for prolonged total hospital stay, and 1.135 (95% CI: 1.014–1.271, *P* = 0.028) for prolonged postoperative hospital stay after full adjustment (Table 3).

**Figure 1:**
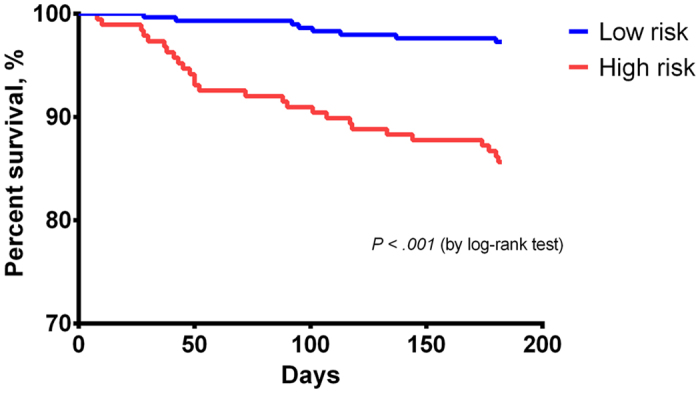
Cumulate 6-month survival rate according to risk stratification based on the Hip-MFS. The Kaplan Meier curve for the cumulative 6-month all-cause survival rates between high risk and low risk Hip-MFS groups. Log-rank test shows significant difference between two groups, statistically.

**Figure 2:**
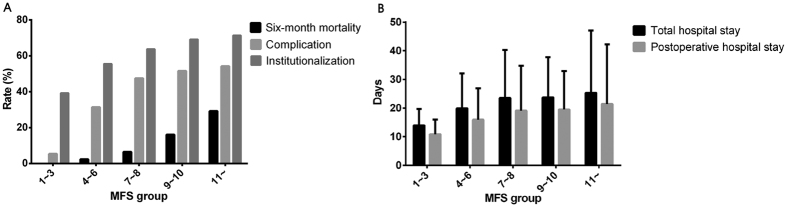
(**A**) Rates of 6-month all-cause mortality, complications, institutionalization. (**B**) Total and postoperative length of hospital stay. Adverse outcomes including 6-month mortality rate, postoperative complication rate, institutionalization rate and length of hospital stay are tend to increase in tandem with the Hip-MFS.

Using the criterion of a score above 8, 117 patients (24.3%) were categorized as high-risk group. The incidence of 6-month all-cause mortality (3.6% vs. 18.8%, *P* < 0.001), postoperative complications (33.5% vs.52.1%, *P* < 0.001), prolonged total hospital stay (19.2% vs. 29.9%, *P* = 0.015), prolonged postoperative hospital stay (22.8% vs. 34.2%, *P* = *0.014*) and institutionalization (56.2% vs. 69.6%, *P* = 0.011) were lower in the low-risk group than in the high-risk group. The cumulative 6-month survival rates of the two groups were statistically significant according to the Kaplan–Meier curve (*P* < 0.001 by log-rank test) (Fig. 1). The median total hospital stay (17 [11–26] vs. 22 [13–29] days, *P* = 0.016) and the median postoperative hospital stay (14 [8–21] vs. 16 [9.5–25] days, *P* = 0.013) were longer in the high-risk group. The 6-month all-cause mortality rate, complication rate, institutionalization rate, and length of hospital stay tended to increase in tandem with the Hip-MFS (Fig. 2A,B). In a multivariate analysis, we confirmed that the high-risk group (Hip-MFS > 8) showed adverse clinical outcomes after hip fracture surgery (Supplementary Table S1A, S1B).

After excluding 50 patients who visited hospital >7 days after trauma, the median waiting time for surgery was 98.1 (69.9–137.2) hours. Predictive values of Hip-MFS on 6-month all-cause mortality were observed in patients with early as well as delayed surgery (Table 4).

Baseline Koval grades were higher in patients with higher Hip-MFS. Furthermore, the deterioration of Koval grade from baseline to 6 months after surgery were significantly greater in patients with higher Hip-MFS (repeated-measure ANOVA P < 0.001) (Supplementary Fig S1).

### Comparing Hip-Multidimensional Frailty Score and Conventional Risk Factors

**Figure 3:**
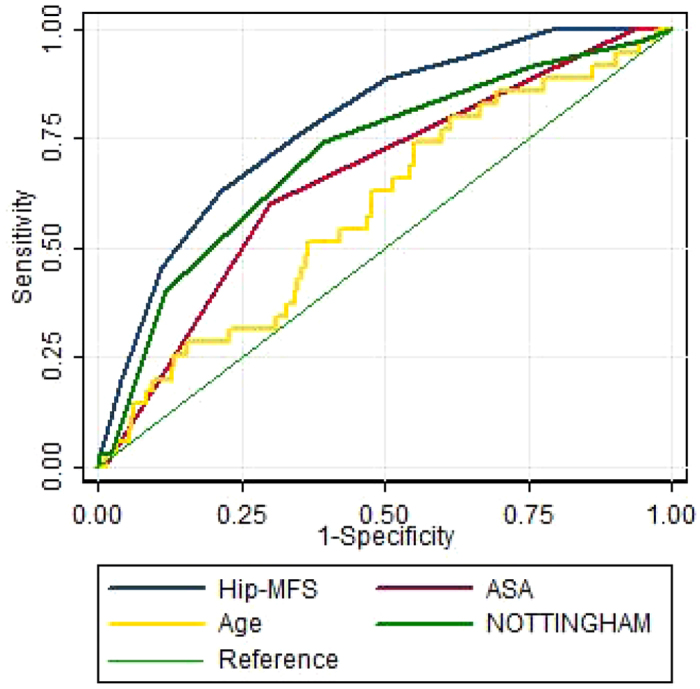
Comparison of area under receiver operating characteristic curve (AUC) for 6-month all-cause mortality rate between Hip-MFS, age, ASA classification and NHFS. Graph showing highest AUC area in Hip-MFS (0.78) than age (0.58), ASA classification (0.67) and NHFS (0.70) with statistical significance with p value of 0.049, 0.001 and 0.033, respectively.

The Hip-MFS predicts 6-month all-cause mortality more accurately than the ASA classification, chronological age and NHFS according to the comparison of AUC (Area under curve) area of ROC curve. AUC area for the predictive model according to Hip-MFS, age, ASA classification and NHFS were 0.784 (95% CI: 0.780–0.787), 0.586 (95% CI: 0.572–0.590), 0.661 (95% CI: 0.657–0.664) and 0.711 (95% CI: 0.707–0.715), respectively. The pairwise comparison of the AUC area were statistically significant different between the Hip-MFS model and the ASA classification (P = 0.002), age (P = 0.004) or NHFS (0.044) (Fig. 3).

## Discussion

In this study, we verified that the Hip-MFS independently predicted 6-month all-cause mortality, 1-year all-cause mortality, postoperative complications, and prolonged total or postoperative hospital stay in elderly hip fracture patients who underwent surgery. Furthermore, the Hip-MFS was expected to be more precise in predicting 6-month all-cause mortality than the conventional ASA classification and the age.

Hip fractures more frequently occur in the elderly population, and they tend to experience complications, decrement of functional ability, institutionalization, and death after surgery more frequently than their younger counterparts because of their unique physiologic vulnerability. Thus, preoperative geriatric assessment markers for frailty, disability, comorbidity, biochemical marker including haemoglobin and albumin, and frailty index are known to predict adverse outcomes in older surgical or trauma patients^25^,^26^,^27^,^28^.

We included gender in the Hip-MFS model because it is known to be uncorrectable and important predictor of outcomes after hip fracture surgery^29^. Recurrent falling and fracture can cause immobilization, complications, institutionalization, mortality, and additional financial burdens. We appraised physical functional capacity by Koval grade instead of ADL/IADL because walking dependency is much easier to evaluate, but strong predictor of mortality in elderly people^30^,^31^.

Previous study has demonstrated that a scoring system such as NHFS could predict mortality after hip fracture^22^. However, Hip-MFS can predict postoperative complications, walking dependency, prolonged hospital stay as well as mortality. Furthermore, Hip-MFS is composed of multiple domains, it signifies the sum of the cumulative deficit and frailty status. Accordingly, Hip-MFS is a better indicator of frailty or vulnerability to stress in elderly people.

Our study has several limitations. First, the patients were recruited retrospectively, collected in only 1 hospital, CGAs were conducted after surgery in 27 patients. Even though most of the component of the Hip-MFS are relatively not change significantly in a short time, retrospectively collected functional assessments of pre-fracture status, such as the Koval grade, are inevitably affected by recall bias because of the patients’ desperate circumstances. In addition, among 1075 patients with hip fracture, we included 490 patients (45.6%) who performed CGA. However, there was no significant difference in baseline characteristics between participants and non-participants of this study (Supplementary Table S2). Furthermore, the 1-year mortality rate (13.9%) was comparable with our previous result (12.2%)^32^. Accordingly, the study samples were representative to a normal hip fracture population of our hospital. Finally, the results were not significantly different when we analysed with the participants who have pre-operative CGA (N = 454).

Patients in the high-risk group tend to visit the hospital less frequently than those in the low-risk group for the follow-up functional assessments, which is a limitation of the hospital-based functional assessment. Because the high-risk patients are expected to achieve less functional recovery but are less evaluated than the low-risk patients, the correlation between the Koval grade and the Hip-MFS could be underestimated.

The Hip-MFS failed to predict institutionalization. Compared with the institutionalization rate (17.3–18.8%) of the previous study for hip fracture patients, the institutionalization rate of the present study (58.6%) was relatively higher^33^,^34^. This high institutionalization rate implies that more patients were discharged to transitional care units than medically needed. Furthermore, the statistical significance of the institutionalization rate could be affected by insurance status, family support, and socioeconomic status.

A strength of our study is the large sample size (N = 481). The Hip-MFS is a geriatric-specific preoperative scoring model, showing a positive correlation with adverse outcomes. This simple dose dependent relationship and identification of high-risk patients can help non-geriatrician orthopaedists, patients, and caregivers easily understand the risks of surgery. Even though the mortality could be affected by time interval to surgery, the discriminative power of Hip-MFS maintained at both early and delayed surgery groups. Risk stratification with Hip-MFS could be helpful to make careful decision and determine the intensity of care during the perioperative period, eventually better clinical outcomes with restricted medical resources. Although the Hip-MFS is the modified model of the MFS, the outcomes of this study consistently show the predictive value of the MFS to predict adverse outcomes after surgery, indicating that the MFS can be widely used for geriatric surgical patients with some modification.

## Additional Information

**How to cite this article:** Choi, J.-Y. *et al*. Prediction of Mortality and Postoperative Complications using the Hip-Multidimensional Frailty Score in Elderly Patients with Hip Fracture. *Sci. Rep.* **7**, 42966; doi: 10.1038/srep42966 (2017).

**Publisher's note:** Springer Nature remains neutral with regard to jurisdictional claims in published maps and institutional affiliations.

## Supplementary Material

Supplementary Information

## Figures and Tables

**Table 1:** Composition of Hip-Multidimensional Frailty Score (Hip-MFS).

Item	Score		
	0	1	2
Sex	Female	Male	NA
Charlson Comorbidity Index	0	1–2	>2
Albumin, g/dL	>3.9	3.5–3.9	<3.5
Koval grade	1	2–6	7
Dementia (MMSE-KC)	Normal	Mild cognitive impairment	Dementia
Risk of falling*	Yes	No	NA
MNA	Normal	Risk of Malnutrition	Malnutrition
Midarm circumference, cm	>27	24.6–27.0	<24.6
Total score: 14, Cut-off values identifying high risk: >8			

^*^The score of Prediction for Falling assessment guide more than 10 (>10) defined as a risk of falling.

MMSE-KC indicates Korean version of the Mini-Mental State Examination; MNA, Mini Nutritional Assessment.

**Table 2:** Comparison of Demographic, Laboratory, and CGA Components by 6-month All-cause Mortality.

	Survival (n = 446)	Death (n = 35)	*P* Values
**Demographic**			
Age (year)	80.3 (6.9)	82.5 (6.8)	0.073
Sex (male/female)	125/321	14/22	0.132
Weight (kg)	52.8 (10.6)	51.3 (8.4)	0.397
Body mass index (kg/m^2^)	21.5 (3.6)	20.5 (3.1)	0.115
ASA class (1/2/3/4)*	29/274/126/4	0/14/21/0	***0.002***
Fracture site (intra/extra)	217/229	18/17	0.752
Anesthesia (general/spinal)	49/397	5/30	0.575
**Laboratory**			
WBCs (×10^3^/μL)	9.5 (3.7)	9.2 (3.1)	0.639
Hemoglobin (g/dL)	11.6 (1.8)	10.6 (2.0)	***0.002***
Platelets (×10^3^/μL)	201.9 (74.6)	217.2 (107.4)	0.413
Creatinine (mg/dL)	0.97 (0.97)	1.62 (1.82)	***0.043***
Protein (mg/dL)	6.5 (0.7)	6.2 (0.7)	***0.018***
Albumin (mg/dL)	3.8 (0.5)	3.4 (0.5)	<***0.001***
AST (IU/L)	24.9 (10.3)	25.8 (10.3)	0.613
ALT (IU/L)	17.4 (17.1)	17.1 (12.2)	0.916
**Comprehensive geriatric assessment**			
Charlson’s comorbidity index	1.2 (1.3)	2.4 (2.0)	***0.001***
Polypharmacy†	348 (78.2%)	28 (80.0%)	0.804
ADL dependency (partial and full)	370 (83.0%)	33 (94.3%)	0.095
IADL dependency	336 (75.3%)	34 (97.1%)	***0.001***
MMSE-KC‡	18.1 (7.6)	13.3 (7.0)	***0.001***
SGDS-K§	4.9 (3.3)	5.0 (3.5)	0.848
Risk of fall (≥10)	327 (73.3%)	33 (94.3%)	***0.004***
MNA	20.7 (5.0)	17.3 (5.1)	***<0.001***
Mid-arm circumference (cm)	23.6 (3.1)	21.6 (4.2)	***0.001***
The Koval grade	2.2 (1.8)	3.4 (2.0)	***0.001***

Data are presented as mean (SD) or number (%).

ADL indicates activities of daily living; ALT, alanine aminotransferase; AST, aspartate aminotransferase; ASA, American Society of Anesthesiologists; CGA, comprehensive geriatric assessment; IADL, instrumental activities of daily living; MMSE-KC, Korean version of the mini-mental status examination; SGDS-K, short form of the Korean Geriatric Depression Scale; MNA, mini nutritional assessment; Nu-DESC, the nursing delirium screening scale; WBC, white blood cell.

^*^Data were missing for 13 patients.

^†^Data were missing for 1 patient.

^‡^Data were missing for 59 patients.

^§^Data were missing for 140 patients.

**Table 3:** Adjusted HRs by Score of Multidimensional Frailty Model for 6-Month Mortality, 1-year Mortality and ORs for Postoperative Complication, Institutionalization and Prolonged Hospital Stay Using Logistic Regression.

	Age adjusted OR (95% CI)	Fully Adjusted OR (95% CI)*
6-month mortality	1.560 (1.333–1.827)^‡^	1.458 (1.210–1.758)^‡^
1-year mortality	1.415 (1.266–1.581)^‡^	1.419 (1.239–1.626)^‡^
Postoperative complications†	1.295 (1.186–1.415)^‡^	1.239 (1.115–1.377)^‡^
Institutionalization	1.166 (1.075–1.265)^‡^	1.078 (0.978–1.191)
Prolonged total hospital stay	1.215 (1.102–1.339)^‡^	1.187 (1.053–1.339)^||^
Prolonged postoperative hospital stay	1.211 (1.104–1.328)^‡^	1.135 (1.014–1.271)^¶^

^*^Adjusted by age, body mass index, white blood cell count, hemoglobin, cholesterol, protein, blood urea nitrogen, creatinine, and American Society of Anesthesiologists class.

^†^A total of 183 patients (38.0%) experienced at least 1 postoperative complication including pneumonia (21 patients), urinary tract infection (20 patients), pulmonary thromboembolism (4 patients), deep vein thrombosis (5 patients), delirium (162 patients), stroke (3 patients), and unplanned ICU admission (18 patients).

^‡^*P* < 0.001, ^||^*P* < 0.01, ^¶^*P* < 0.05. CI, indicates confidence interval; ICU, intensive care unit; HR, hazard ratio; OR, odds ratio.

**Table 4:** Comparison of 6-month mortality according to Hip-MFS risk group stratified by time to surgery.

	Low risk Hip-MFS (Hip-MFS ≤ 8) (n = 314)	High risk Hip-MFS (Hip-MFS > 8) (n = 117)	*P* Values§
Death within 6 months for early surgery cohort (≤120 h from admission)*	1/38 (2.6%)	3/6 (50.0%)	<0.001
**Death with 6 months for delayed surgery cohort (>120 h from admission)**†	11/289 (3.8%)	16/98 (16.3%)	<0.001

Total 431 patients were included for analysis.

^*^44 patients were included for analysis.

^†^387 patients were included for analysis.

^§^P value by log-rank test.
