# Supplementary material for: Prediction of Mortality and Postoperative Complications using the Hip-Multidimensional Frailty Score in Elderly Patients with Hip Fracture
Source: Sci Rep. 2017 Feb 24;7:42966. doi: 10.1038/srep42966 (PMC5324046; doi:10.1038/srep42966)
Supplement: Supplementary Information [file srep42966-s1.pdf]

Prediction of Mortality and Postoperative Complications using the Hip-Multidimensional Frailty Score in Elderly Patients with Hip Fracture

Jung-Yeon Choi, MD,[1] Kwan-Jae Cho, MD,[2] Sun-wook Kim, MD,[1,3] MSc Sol-Ji Yoon, MD,[1] Min-gu Kang, MD,[1] Kwang-il Kim, MD, PhD,[1,3,*] Young-Kyun Lee, MD, PhD,[4] Kyung-Hoi Koo, MD, PhD,[2,4] & Cheol-Ho Kim, MD, PhD[1,3]

[1]Department of Internal Medicine, Seoul National University College of Medicine, Seoul, Republic of Korea. [2]Department of Orthopedic Surgery, Seoul National University College of Medicine, Seoul, Republic of Korea. [3]Department of Internal Medicine Seoul National University Bundang Hospital, Seongnam, Republic of Korea. [4]Department of Orthopedic Surgery, Seoul National University Bundang Hospital, Seongnam, Republic of Korea.

SUPPLEMENTARY TABLE 1-A. Adjusted HRs by Risk of Multidimensional Frailty Model for 6-month and 1-year all-cause mortality Using Cox Regression

	Age adjusted HR (95% CI)	Fully Adjusted HR* (95% CI)
6-month mortality	5.428 (2.713 – 10.858) †	3.910 (1.798 – 8.505) ‡
1-year mortality	3.589 (2.211 – 5.825) †	3.393 (1.929 – 5.968) †

*Adjusted by age, body mass index, white blood cell count, hemoglobin, cholesterol, protein, blood urea nitrogen, creatinine, and American Society of Anesthesiologists class.

†$P < 0.001$, ‡$P = 0.001$.

CI indicates confidence interval; HR, hazard ratio

SUPPLEMENTARY TABLE 1-B. Comparison of Postoperative Complications, Institutionalization and Prolonged Hospital Stay according to Hip-MFS risk group.

	**Low-risk Hip-MFS (Hip-MFS ≤ 8) (n=364)**	**High risk Hip-MFS (Hip-MFS > 8) (n=117)**	**P Values***
Postoperative complications[†]	122 (33.5%)	61 (52.1%)	< ***0.001***
Institutionalization*	204 (56.2%)	78 (69.6%)	***0.011***
Prolonged total hospital stay	70 (19.2%)	35 (29.9%)	***0.015***
Prolonged postoperative hospital stay	83 (22.8%)	40 (34.2%)	***0.014***

P value comparing high-risk Hip-MFS group and low-risk Hip-MFS group by χ^2 or Fisher's exact test.

*Data not included 6 patients because of in-hospital mortality..

SUPPLEMENTARY TABLE 2. Comparison of Baseline Demographic Characteristics between Participants and Non-participants of the study.

	Participants (n = 481)	Non-participants (n = 594)	*P* Values
**Demographic**			
Age (year)	80.5 (6.9)	80.3 (7.0)	0.620
Sex (male/female)	139/342	158/436	0.402
Weight (kg)	52.7 (10.5)	53.4 (11.1)	0.297
Body mass index (kg/m^2)	21.4 (3.6)	21.7 (3.9)	0.152
ASA class (1/2/3/4)*	29/288/147/4	32/355/182/7	0.918
Fracture site (intra/extra)	235/246	280/314	0.575
HTN	328 (68.2%)	386 (65.0%)	0.268
DM	163 (33.9%)	201 (33.8%)	0.986
History of MI	57 (11.9)	65 (10.9%)	0.641
Heart Failure	11 (2.3%)	21 (3.5%)	0.231
History of CVA	100 (20.8%)	105 (17.7%)	0.196
Chronic Kidney Disease	36 (7.5%)	54 (9.1%)	0.344
Chronic Liver Disease	22 (4.6%)	27 (4.5%)	0.982

Data are presented as mean (SD) or number (%).

ASA indicates American Society of Anesthesiologists; HTN, Hypertension; DM, Diabetes mellitus; MI, Myocardial infarction;

*Data were missing for 30 patients.

Supplementary Figure S1. Distribution of the patient's Koval grades according to Hip-MFS.

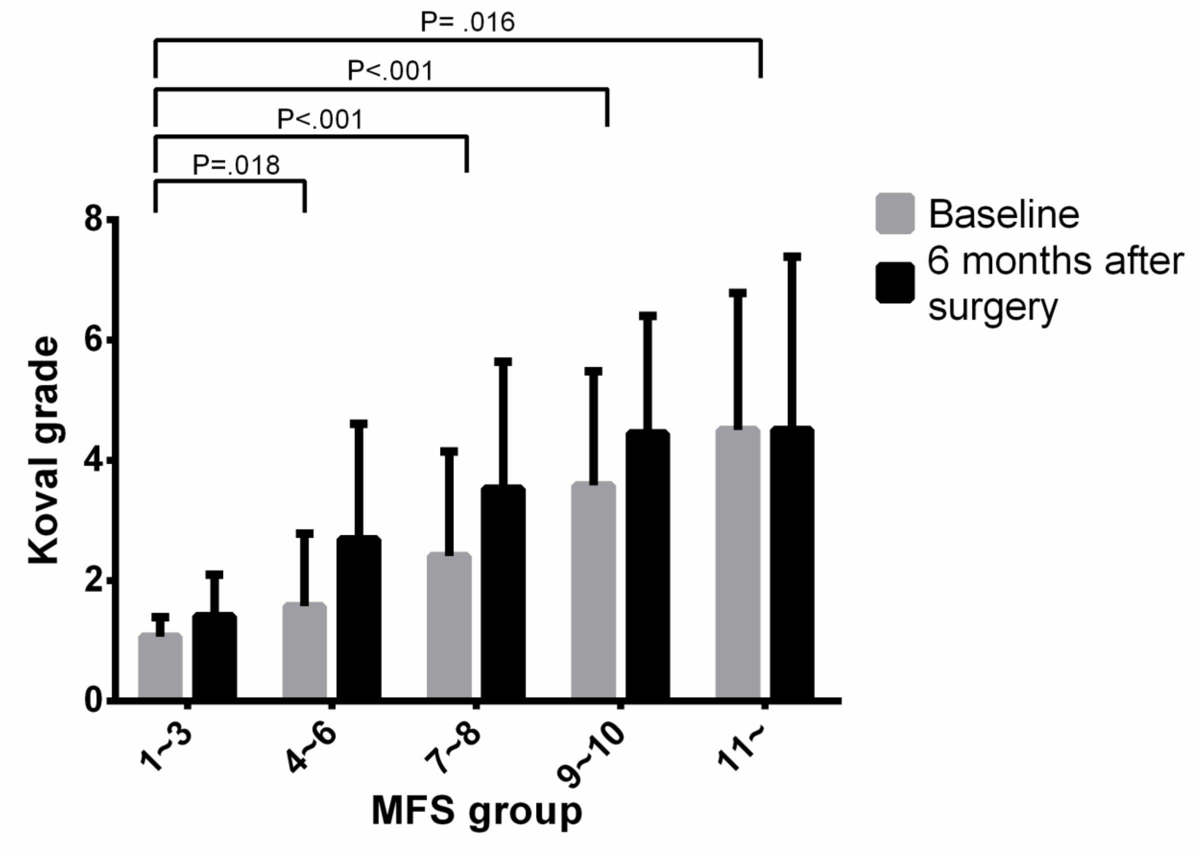

Supplementary Figure S1. Distribution of the patient's Koval grades according to Hip-MFS.

Plots of mean ± standard deviation of the Koval grade according to the risk of Hip-MFS at baseline and 6 months after hip fracture surgery. Bars denotes means and horizontal bars denotes standard deviations. P-values shown denote statistical significance of between-group deterioration of Koval grade from baseline and 6 months after surgery by post hoc analysis. The P values of the post hoc analysis between the group of the Hip-MFS from 1 to 3 and group of the Hip-MFS from 4 to 6 or 7 to 8 or 9 to 10 or more than 11 were 0.018, < 0.001, < 0.001 and 0.016, respectively.
